# Autonomic Nervous System Dysfunction in Diabetic Patients After Myocardial Infarction: Prognostic Role of the Valsalva Maneuver

**DOI:** 10.3390/medicina62010096

**Published:** 2026-01-01

**Authors:** Nikola Marković, Maša Petrović, Vasko Žugić, Sulin Bulatović, Milovan Bojić, Branislav Milovanović

**Affiliations:** 1 Institute for Cardiovascular Diseases “Dedinje”, 15300 Belgrade, Serbia; 2 School of Medicine, University of Belgrade, 11000 Belgrade, Serbia

**Keywords:** autonomic nervous system, diabetes mellitus, myocardial infarction, Valsalva maneuver, heart rate variability, prognosis

## Abstract

*Background and Objectives*: Diabetes mellitus (DM) is a major risk factor for cardiovascular diseases (CVD), including acute myocardial infarction (MI), and is frequently associated with cardiac autonomic neuropathy (CAN). Post-MI autonomic dysfunction contributes to adverse outcomes, but data on prognostic markers in diabetic patients remain limited. This study aimed to (1) compare autonomic nervous system (ANS) function between patients with MI and DM (MI/DM), MI without DM, and DM without MI; (2) assess differences in MI/DM patients based on survival status; and (3) identify prognostic factors for all-cause mortality in diabetic patients following MI. *Materials and Methods*: This retrospective–prospective study included 375 patients: 93 MI/DM, 229 MI, and 53 DM. MI patients were treated with fibrinolytic or conservative therapy. All participants underwent cardiovascular reflex tests (CARTs) and 24 h Holter ECG with heart rate variability (HRV) analysis; DM patients without MI were tested in an outpatient setting. The primary endpoint was all-cause mortality during a median follow-up of 38 months. Univariable and multivariable Cox regression analyses were performed to determine mortality predictors. *Results*: Autonomic dysfunction was prevalent in all groups, with MI/DM patients showing the most pronounced impairment, particularly in parasympathetic function. MI/DM patients had significantly lower SDNN values and higher prevalence of definite parasympathetic dysfunction than other groups. In the MI/DM group, abnormal Valsalva maneuver (VM) was more frequent among non-survivors. Multivariable analysis identified abnormal VM and NSTEMI as predictors of overall mortality. *Conclusions*: Diabetic patients after MI exhibit the most severe autonomic impairment, predominantly parasympathetic, which may contribute to their increased cardiovascular risk. In this high-risk group, abnormal VM and NSTEMI presentations independently predict long-term mortality. Assessment of autonomic function, particularly VM, may provide valuable prognostic information and aid in risk stratification.

## 1. Introduction

Cardiovascular diseases (CVDs) remain one of the leading causes of morbidity and mortality worldwide, with acute coronary syndrome (ACS) frequently constituting the initial clinical manifestation [1]. It is estimated that nearly two million deaths among both women and men are attributable to CVD, of which approximately 40–45%, depending on sex, result from ACS [1]. Although contemporary ACS guidelines emphasize percutaneous coronary intervention (PCI) as the primary therapeutic strategy, the identification of prognostic factors associated with long-term mortality continues to be a critical area of research. Numerous studies have highlighted a wide range of potential predictors, demonstrating variability in their relative clinical importance [2,3].

Among established CVD risk factors, diabetes mellitus (DM) is particularly notable. DM markedly increases the risk of developing nearly all major forms of cardiovascular pathology, including ACS, atrial fibrillation (A.fib), heart failure (HF), stroke, and both aortic and peripheral artery disease (PAD) [4]. Patients with DM often present with atypical or nonspecific ACS symptoms, contributing to delayed diagnosis and consequently delayed initiation of therapy [1]. Furthermore, diabetic patients commonly exhibit more extensive coronary artery disease at presentation, such as multivessel involvement or left main coronary artery disease, and frequently receive less optimal care during both the acute and post-ACS phases: factors that together contribute to poorer long-term outcomes [1,5].

Beyond the classical pathophysiological mechanisms linking DM to CVD (i.e., atherosclerosis, endothelial dysfunction, impaired vascular smooth muscle reactivity, and a prothrombotic milieu), DM is also a major cause of cardiac autonomic neuropathy (CAN) [6]. CAN is a highly prevalent yet often underdiagnosed microvascular complication, with reported prevalence ranging from 10% to 70% [6]. Importantly, individuals with established diabetic CAN have a five-year mortality rate of 25% to 50% [6]. CAN is further recognized as a contributor to multiple cardiovascular manifestations, including HF, A.fib, and ischemic heart disease, particularly silent myocardial ischemia [4,7,8].

Conversely, cardiac diseases can induce both anatomical (primary) and functional (secondary) alterations in cardiac autonomic function [9]. Following acute myocardial infarction (MI), the cardiac autonomic nervous system undergoes a series of structural and neurochemical changes characterized by increased sympathetic drive, heterogeneous patterns of denervation and hyperinnervation, neuronal hypertrophy, altered neurotransmitter signaling within the stellate ganglion, reduced afferent signaling from infarcted myocardium, and a pronounced loss of intracardiac neurons expressing choline acetyltransferase [9,10,11].

Previous research has identified several autonomic biomarkers of prognostic significance after MI, including heart rate variability (HRV) indices and deceleration capacity (DC) [12,13]. Abramkin et al. further demonstrated the prognostic value of the Valsalva maneuver (VM) in predicting sudden cardiac death (SCD) following MI [14]. In addition, Ren et al. reported lower HRV parameters (including SDNN, SDANN, and high-frequency (HF) power) in post-MI patients with DM compared to those without DM [15]. Similarly, Stoičkov et al. found that diabetic patients exhibited significantly reduced time-domain HRV indices and more frequent, complex ventricular arrhythmias six months after acute MI compared with their non-diabetic counterparts [16].

The aims of this study are (1) to evaluate differences in autonomic nervous system (ANS) function among three groups: (a) patients with acute MI and DM, (b) patients with acute MI without DM, and (c) patients with DM without a history of MI. (2) To assess differences within the group of patients with both MI and DM based on survival status at the end of follow-up. (3) To identify prognostic factors associated with overall mortality in patients with DM following acute MI.

## 2. Materials and Methods

This retrospective—prospective study included 375 patients, of whom 322 were admitted between 2003 and 2013 to the Coronary Care Unit of Clinical Hospital Center Bežanijska Kosa, Belgrade, Serbia, due to acute myocardial infarction, and 53 DM patients without previous MI (DM group). Of the 322 patients with MI, 93 (28.9%) had DM and were classified as the MI/DM group, while the remaining 229 patients without DM were categorized as the MI group. Patients with MI (both groups) were initially treated with fibrinolytic therapy/conservative treatment, mainly depending on the time of presentation and/or contraindication for fibrinolytic therapy [17,18].

Inclusion criteria for both MI groups were acute ischemic heart disease confirmed by (1) characteristic clinical presentation (chest pain or chest pain–equivalent symptoms); (2) elevated cardiac biomarkers (high-sensitivity cardiac troponin); and (3) ST-segment changes consistent with guideline recommendations [1]. Diagnosis of DM was confirmed by an endocrinologist.

All patients received standard-of-care medical therapy in accordance with contemporary clinical guidelines, including beta-blockers, calcium channel blockers, angiotensin-converting enzyme inhibitors, and other indicated cardiovascular medications. Pharmacological therapy was not discontinued on the day of autonomic testing in any study group. In patients with recent myocardial infarction, interruption of guideline-directed medical treatment was not considered clinically appropriate. In patients without myocardial infarction, ongoing therapy was likewise maintained in order to assess autonomic function under real-world clinical conditions and to reflect routine clinical practice. Exclusion criteria included: terminal-stage malignancy; severe respiratory or renal failure; pacemaker implantation; persistent atrial fibrillation or atrial flutter; and any non-sinus rhythm at the time of cardiovascular autonomic reflex testing and Holter ECG recording. Patients who were hemodynamically unstable on the 21st day after myocardial infarction, or who had severe aortic stenosis, hypertrophic cardiomyopathy with left ventricular outflow tract obstruction, or other conditions preventing reliable autonomic testing were excluded. Patients with primary autonomic neuropathies (e.g., Parkinson’s disease, multiple system atrophy, pure autonomic failure), as well as cases with incomplete or inaccurate data, were also excluded.

The study adhered to the principles of the Declaration of Helsinki and received initial approval from the Scientific Ethical Committee of the University Clinical Center “Bežanijska Kosa” (protocol code 1039/3; approval date 12 April 2011). It was subsequently re-approved by the Ethics Committee of the Institute for Cardiovascular Diseases “Dedinje” (protocol code 6470; re-approval date 11 December 2024). The study was supported by grant TP 32040 from the Ministry of Education, Science, and Technological Development of the Republic of Serbia.

Data were extracted from the medical records of all eligible patients. Collected information included clinical characteristics at the time of admission, relevant diagnostic tests, and imaging performed during hospitalization. The analyzed variables covered: (1) demographic data (age and gender), (2) treatment approach (fibrinolytic/conservative therapy (applicable to MI groups)), (3) medical history of previous cardiovascular events, (4) infarct localization (for MI patients) (Anteroseptal (AS), inferoposterior (IP), Non—ST elevation MI (NSTEMI) and other localization (right ventricle, lateral infarction), (5) Clinical status at admission (for MI groups), (6) left ventricular ejection fraction (EF)—assessed via echocardiography 24 h after admission using the Simpson Biplane method (for MI patients), (7) 24 h Holter ECG monitoring and (8) CART, both performed on the 21st day of hospitalization for MI patients and ambulatory for DM patients without MI [19].

Twenty-four-hour ambulatory ECG recordings were obtained using a 3-lead electrocardiograph (Cardioscan, D.M.S., Nevada, USA) and analyzed by experienced physicians. All recordings were manually reviewed, corrected, and prepared for subsequent analysis. Heart rate variability (HRV) measures were derived in both time and frequency domains using system software. Time-domain metrics included: standard deviation of normal RR intervals (SDNN), standard deviation of 5 min average NN intervals (SDANN), and root mean square of successive differences (RMSSD). Frequency-domain parameters included low-frequency (LF; 0.04–0.15 Hz), high-frequency (HF; >0.15 Hz), and the LF/HF ratio.

The CART, as described by Ewing, consists of five tests designed to assess the function of the autonomic nervous system [20]. Two of these tests evaluate sympathetic function: the Hand Grip Test (HGT) and the Blood Pressure Response to Standing Test (OH). Three tests assess parasympathetic function: the VM (assessed by Valsalva ratio (VR)), Heart Rate Response to Deep Breathing (HRB), and Heart Rate Response to Standing (HRS). According to Ewing’s criteria, test results are categorized as normal, borderline, or abnormal [20]. Each test result is assigned a numerical value: normal (0), borderline (1), and abnormal (2) [21]. The scores for all tests are summed up, with the total score ranging from 0 to 10. If at least one sympathetic function test result is abnormal, it indicates sympathetic dysfunction. One abnormal parasympathetic function test suggests initial parasympathetic dysfunction (PD), while two or more abnormal tests indicate definite parasympathetic dysfunction [20]. Complete autonomic dysfunction (CAD) is defined by the presence of both sympathetic dysfunction and either initial or definite parasympathetic dysfunction. According to Bellavere et al., borderline outcomes are often attributable to improper test execution, and they advocate for retesting in such cases [21]. In the context of this study, borderline findings were classified as normal.

Following hospital discharge, patients with DM were monitored for clinical outcomes of interest, specifically overall mortality. Follow-up information was obtained from medical records and structured telephone interviews to determine survival status after the index hospitalization.

### Statistical Analysis

Results are presented as mean and standard deviation (SD), median (Mdn) with 25–75% interquartile range (IQR), or as a count, depending on the data type. Continuous data were tested for normality using the Kolmogorov–Smirnov test for normality, as well as normal and detrended normal Q–Q plot. Groups are compared using parametric (ANOVA with post hoc Bonferroni test) and nonparametric tests: (1) Kruskal–Wallis test, followed by Dunn–Bonferroni correction test for numeric and (2) Chi Square and Fisher Exact test for nominal/ordinal data. The level of significance was set at *p* < 0.05 or <0.016, depending on the number of groups being compared.

Kaplan–Meier analysis was employed to estimate the survival function of participants, with overall mortality as the primary endpoint. Survival time was calculated from the date of the first visit until the occurrence of the specified outcome. Continuous variables were categorized using median values or pre-established cutoff points. To examine the relationship between predictors and time to event, univariable Cox proportional hazards regression was applied. Hazard ratios (HRs) with 95% confidence intervals (CIs) were calculated for each predictor. Variables with a *p*-value < 0.05 in the univariable analysis were considered for inclusion in the multivariable model. For variables that could be both continuous and categorical, continuous versions were used in the final model, as they provided more precise estimates of the predictor-outcome relationship. A multivariable Cox regression analysis was then conducted using forward conditional selection, starting with the most significant predictor identified in the univariable analysis. This process involved iteratively adding variables that contributed significantly to the model (*p* < 0.05), while excluding those with a *p*-value > 0.05. All statistical analyses were performed using SPSS version 26.0.

## 3. Results

This study included 53 patients with DM and no prior history of MI (DM group), 322 patients presenting with MI, of whom 93 had DM (MI/DM group), and 229 did not (MI group). All MI patients were treated either with fibrinolytic therapy or conservative management. In the MI/DM group, after a median follow-up of 38 months (interquartile range: 16–58 months), 16 patients (17.2%) died from all causes.

Table 1 presents the baseline characteristics, clinical status at admission, and left ventricular ejection fraction (EF) of the study population. The MI/DM group was significantly older than both the MI and DM groups. Although no other statistically significant differences were observed across groups, male sex was predominant in all three cohorts. Approximately 30% of MI patients in both the MI and MI/DM groups received fibrinolytic therapy. More than 60% of patients in each MI group were classified as Killip class I at admission. The mean EF values in both MI groups exceeded 40%.

In Table 2, the results of the CART assessments are presented. Abnormal HRB, as well as definite DP, were significantly more frequent in the MI/DM group compared with the other two groups. The MI/DM group also demonstrated a higher overall autonomic neuropathy (AN) score than the MI group. No additional statistically significant differences were observed across the groups.

In Table 3, the results of 24 h Holter ECG monitoring are presented. The mean heart rate (HR) was significantly lower in both MI groups compared with the DM group. SDNN values were lowest in the MI/DM group compared to the other two groups. The MI/DM group exhibited the highest SDANN values. Although RMSSD, LF, and HF values were lowest in the MI/DM group, these differences did not reach statistical significance. Across all three groups, mean LF/HF ratios were above 3.5.

Table 4 presents the results of the Cardiovascular Reflex Tests in the MI/DM group stratified by survival status at the end of follow-up. Abnormal VM responses were significantly more frequent among MI/DM patients who died compared with those who survived. Although abnormal HRB and definite DP were also more prevalent in the non-surviving group, these differences did not reach statistical significance.

In Supplementary Table S1, the baseline characteristics, clinical status at admission, and EF values of the MI/DM group (stratified by survival status at the end of follow-up) are presented. No statistically significant differences were observed between survivors and non-survivors.

Supplementary Table S2 displays the results of 24 h Holter ECG monitoring in the MI/DM group according to survival status. Although HRV parameters were generally higher among surviving patients, none of these differences reached statistical significance.

Table 5 presents the factors associated with all-cause mortality in patients with DM following MI. In the univariable analyses, only three variables emerged as significant predictors: abnormal VM, Killip class III, and NSTEMI as the index MI type. In the final multivariable model, NSTEMI remained strongly associated with overall mortality, and abnormal VM was also a significant predictor, with a hazard ratio approaching 4.

For the purposes of both univariable and multivariable modeling and given that none of the patients with MI localized to “other” regions or without parasympathetic dysfunction experienced the outcome of interest, these categories were merged with the inferoposterior (IP) MI group and the early parasympathetic dysfunction category, respectively. Ventricular tachycardia (VT), ventricular fibrillation (VF), and A.fib were not included in the regression analyses because no patients with these arrhythmias experienced the outcome of interest (Supplementary Table S3).

Due to the large number of evaluated variables and the absence of statistical significance for many of them, only selected predictors are presented in the main manuscript. The complete model, including all covariates, is provided in Supplementary Table S3, while Supplementary Table S4 lists all examined variables with their corresponding *p*-values.

In Supplementary Table S5, the baseline characteristics, clinical status at admission, and EF values of the MI/DM group stratified according to VM results are presented. No statistically significant differences were identified.

## 4. Discussion

In this study, evidence of autonomic dysfunction was observed across all three study groups, with the most pronounced impairment identified in patients with DM following MI. This dysfunction was predominantly characterized by withdrawal of parasympathetic tone. Furthermore, within the MI/DM group, survival analysis demonstrated that abnormal VM responses and MI localization were significant prognostic indicators of long-term mortality.

As shown in Table 1, the majority of participants were older than 50 years, with the MI/DM group being significantly older than both the MI-only and DM-only groups. Male sex predominated across all three cohorts. These findings are consistent with large epidemiological studies such as that by Milcent et al., which reported that men more frequently experience MI, with mean ages of 63 years in men and 75 years in women [22]. Similarly, Bouisset et al. found that patients with both DM and MI tend to be older and exhibit a higher prevalence of heart failure than those without DM [23]. Older age is a well-recognized risk factor for autonomic neuropathy, particularly in individuals with DM, in whom CAN strongly correlates with age and other microangiopathic complications [24]. Nonetheless, in the present study, age did not predict overall mortality in the MI/DM group (Table 5 and Tables S3–S5), nor did age differ significantly between survivors and non-survivors.

CART testing revealed marked differences in autonomic function among groups (Table 2). CART was originally developed to assess autonomic neuropathy in diabetic patients [20,21], and in this study, abnormal HRB findings differed significantly across all three groups, with the highest prevalence in the MI/DM group and the lowest in the DM-only group. HRB reflects heart rate variation during inspiration and expiration, primarily mediated by the Bainbridge (atrial) reflex [25]. Raje et al. demonstrated that HRB may serve as an initial screening tool for CAN in type 2 DM, with confirmatory testing used for diagnostic classification [26]. In the present study, HRB findings aligned with the distribution of definite DP, which was most prevalent in the MI/DM group and differed significantly among all groups (Table 2).

The pathophysiological sequence of autonomic dysfunction in DM is well established: the longest nerves, including those of the parasympathetic system—particularly the vagus nerve—are affected earliest, while sympathetic impairment generally occurs later and manifests clinically as OH [6]. As shown in Table 2, OH was present in 11–19% of DM patients, depending on group allocation. Interestingly, even MI patients without DM exhibited a higher prevalence of definite parasympathetic dysfunction than the DM-only group. This observation is consistent with the known autonomic perturbations during and after ACS, characterized by sustained sympathetic activation, parasympathetic withdrawal, impaired afferent myocardial signaling, and neurochemical changes in autonomic ganglia [9,10,11]. When combined with the established autonomic impairment in DM, it is plausible that the MI/DM group demonstrated the greatest degree of dysfunction due to cumulative autonomic imbalance from both pathological states.

Analysis of HRV parameters further supports this interpretation. As shown in Table 3, HR was significantly higher in the DM-only group. This is likely attributable to sympathetic overactivity and the fact that beta-blockers, a standard component of post-MI therapy, were used extensively in both MI groups, but not routinely in DM-only patients unless specific cardiovascular indications were present [4]. SDNN values were lowest in the MI/DM group and highest in the DM-only group (Table 3). Rueda-Ochoa et al. identified low SDNN values as one of the most reliable predictors of cardiovascular mortality following ACS, while Nolan et al. reported a 5.5% increase in one-year mortality in patients with SDNN values below 100 ms after ACS [27,28]. Reduced SDNN is also considered a reflection of sympathetic dominance, but LF/HF ratios (commonly used indicators of sympathovagal balance) were above 3 in all three groups, indicating pronounced sympathetic overdrive (Table 3) [29]. In addition, values of HF and RMSSD (two primary HRV parameters for assessing parasympathetic function) were lowest in the MI/DM group, although without reaching statistical significance [30]. As previously noted, both Ren et al. and Stoičkov et al. verified lower SDNN and SDANN values in patients with MI/DM compared to those with MI alone, which is consistent with the findings of this study [15,16]. Considering the results of 24 h HRV analysis and the findings from CART testing, it can be concluded that a certain degree of autonomic dysfunction is present in all three groups, primarily involving parasympathetic impairment. However, these alterations appear to be most pronounced in the MI/DM group, and may therefore significantly contribute to their elevated cardiovascular risk.

**Figure 1:**
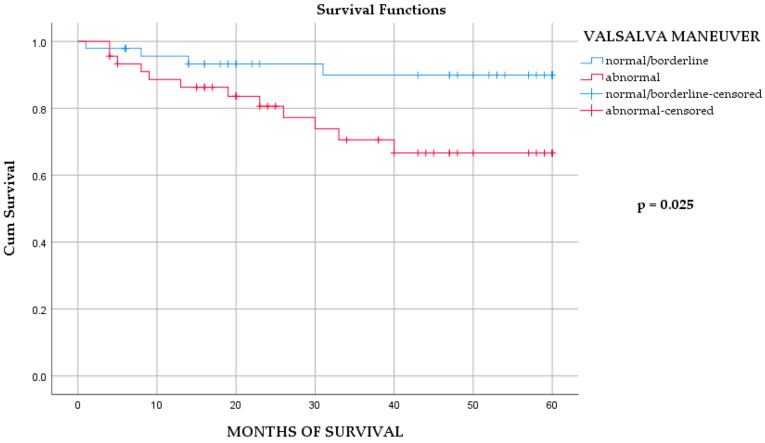
The Kaplan–Meier survival analysis of overall mortality in MI/DM group, according to Valsalva maneuver outcomes. Log-rank test *p* = 0.025. Number at risk at months 0, 20, 40, and 60: normal/borderline = N_0_, 32, 8, 0; abnormal = N_0_, 30, 17, 0.

Within the MI/DM group, survival analysis revealed that abnormal VM was the only autonomic parameter significantly associated with mortality (Table 4, Tables S1 and S2; Figure 1). In the multivariable model, abnormal VM remained a significant predictor alongside NSTEMI (Table 5). Importantly, VM results were not associated with demographic or clinical characteristics, EF, or admission status (Supplementary Table S5), suggesting that abnormal VM reflects intrinsic autonomic dysfunction rather than confounding clinical variables.

This finding is particularly noteworthy given that HRB was the only test to significantly differ across all three groups (Table 2). Although both VM and HRB are used to assess parasympathetic function, they rely on distinct underlying physiological mechanisms. While Jha et al. demonstrated that VM initially triggers sympathetic activation, Low and Benarroch emphasized that when evaluated using the Valsalva ratio—as performed in this study—VM reflects the cardiovagal reflex and corresponds to the vagal component of the baroreceptor arc [31,32]. In contrast, HRB is primarily mediated by the atrial (Bainbridge) reflex, indicating that the reflex pathways involved in the two tests are different [25]. Nevertheless, according to Cui et al., both the Bainbridge and baroreceptor reflexes can be co-activated during deep breathing [33]. The Bainbridge reflex tends to predominate at lower heart rates, whereas at higher heart rates, the increased venous return more strongly engages the baroreceptors [25,34]. This physiological interplay may help explain why, among all autonomic function tests analyzed, only VM emerged as a statistically significant predictor. Moreover, a study by Abramkin et al. that included both VM and HRB found that only VM was a significant predictor of SCD in patients with sinus rhythm and without advanced HF [14]. Therefore, although Raje et al. proposed HRB as a useful preliminary tool for screening CAN in patients with type 2 DM, the findings of this study support the potential use of VM as a prognostic marker for all-cause mortality in diabetic patients following MI [26].

An important factor that may have influenced the study results relates to the treatment strategies applied in the study population. PCI is currently considered the gold standard for the management of acute MI, being associated with lower mortality and fewer major adverse events compared with fibrinolytic or conservative therapy [1,35,36]. Nevertheless, fibrinolytic and conservative treatment approaches remain clinically relevant in settings with limited access to PCI or delayed patient presentation [35,36]. Following acute myocardial infarction, autonomic imbalance develops as part of the post-infarction remodeling process and appears to occur largely independently of the reperfusion strategy [9,10,11]. The prognostic value of autonomic markers has been confirmed in the contemporary PCI era, primarily through heart rate variability and DC analyses [12,13]. However, modern reperfusion and secondary prevention strategies may modify the extent and time course of autonomic dysfunction and alter the pattern of clinical outcomes in PCI-treated cohorts, which should be considered when interpreting the present findings.

An additional aspect that should be considered when interpreting the present findings is background pharmacological therapy. All patients were treated according to contemporary guideline-directed medical therapy, including beta-blockers, calcium channel blockers, and other cardiovascular medications, which are known to influence autonomic regulation [37,38]. Autonomic assessment was therefore performed under ongoing treatment conditions to reflect routine clinical practice. This approach may have contributed to variability in autonomic indices, but it allows interpretation of the results in a real-world clinical context.

NSTEMI emerged as a significant predictor of mortality in the final multivariable model. This finding should be interpreted cautiously, as NSTEMI in patients with diabetes mellitus often reflects a more complex clinical substrate, including a higher burden of comorbidities, diffuse and multivessel coronary artery disease, and recurrent or prolonged ischemic episodes, rather than the infarct type alone [39,40]. Prior studies have shown that improvements in both short- and long-term outcomes have been less pronounced for NSTEMI compared with STEMI [39], supporting the concept that NSTEMI frequently represents a marker of advanced and heterogeneous ischemic heart disease, particularly in diabetic populations.

Although Killip class III was associated with mortality in univariable analysis, it did not remain significant in the multivariable model (Table 4). Diabetes is known to increase the risk of cardiogenic shock and adverse outcomes [41,42,43]; however, no patients in the MI/DM group presented with cardiogenic shock at admission (Table 1). This may explain the lack of significance in our adjusted models.

### Study Limitations

Several limitations should be acknowledged. First, the sample size, particularly with regard to the number of outcome events in the MI/DM subgroup (n = 16), limits statistical power and reduces the precision of effect estimates; therefore, non-significant findings for several parameters may reflect insufficient power rather than the absence of true associations. Second, although a multivariable Cox model was performed using a parsimonious forward conditional approach, the low event count entails a risk of model overfitting, which is also suggested by the wide confidence interval for NSTEMI; thus, multivariable results should be interpreted as exploratory and hypothesis-generating. In this context, although three variables were significant in univariable analysis, only two remained in the final multivariable model following the selection procedure (events per variable ratio = 8). Third, the treatment context reflects the period and local availability of reperfusion strategies; since contemporary guideline-recommended MI management relies on PCI, our findings require confirmation in larger PCI-treated MI cohorts. Fourth, the study lacked detailed coronary angiography and revascularization data (e.g., culprit lesion characteristics, disease extent, and completeness of revascularization), which could influence prognosis and potentially interact with autonomic markers. Fifth, diabetes-specific clinical data were not available in sufficient detail (e.g., duration of diabetes, glycemic control indices such as HbA1c, microvascular complications, and antidiabetic therapy), all of which are known to influence the development of cardiac autonomic neuropathy and clinical prognosis, thereby limiting mechanistic interpretation of the present findings. Although this study was not designed to evaluate the impact of pharmacological therapy on survival, future investigations should include detailed medication-related data. Also, we did not compare our autonomic assessment with additional established markers (e.g., DC), which should be incorporated in future studies to improve risk stratification. Finally, borderline cardiovascular autonomic reflex test results were classified as normal, which may have led to an underestimation of autonomic dysfunction, particularly in patients with diabetes mellitus. However, this approach was consistent with prior methodological recommendations and was considered appropriate given the limited number of outcome events and the absence of events in some potential subgroups.

## 5. Conclusions

Diabetes mellitus is one of the most important risk factors for cardiovascular disease, particularly ischemic heart disease and acute coronary syndrome. In addition to classical pathophysiological pathways, diabetes also contributes to cardiovascular risk through the development of cardiac autonomic neuropathy. In this study, varying degrees of autonomic dysfunction (predominantly parasympathetic impairment) were observed across all three groups: diabetic patients following myocardial infarction, patients with MI without diabetes, and diabetic patients without MI. These abnormalities were most pronounced in the MI/DM group, suggesting a cumulative detrimental effect of diabetes and myocardial infarction on autonomic regulation. Among diabetic patients following myocardial infarction, non-ST-elevation myocardial infarction and abnormal Valsalva maneuver responses, with impaired cardiovagal function and baroreflex sensitivity, increased long-term mortality was observed. Although these findings should be interpreted with caution due to the limited number of outcome events, they underscore the potential clinical relevance of autonomic function assessment in this high-risk population. Further studies in larger, contemporary PCI-treated cohorts are warranted to confirm the prognostic value of autonomic testing and to define its role in risk stratification and long-term management after myocardial infarction.

## Figures and Tables

**Table 1:** Basic characteristics, clinical status upon admission and EF of the study population.

	1. MI/DM N = 93	2. MI N = 229	3. DM N = 53	*p* Value
**Basic characteristics of study population**				
Age (yrs.) (mean ± SD)	63.3 ± 8.6 ^2,3^	58.9 ± 9.9 ^1,3^	53.6 ± 10.7 ^1,2^	<0.001 ^a^
Male (n,%)	56 (60.2%)	158 (69%)	31 (58.5%)	0.171 ^b^
Previous MI (n,%)	13 (14%)	27 (11.8%)		0.590 ^b^
**Treatment strategy**				
Fibrinolytic therapy	27 (29%)	76 (33.2%)		0.469 ^b^
**Clinical status at admission**				
AS MI (n,%)	38 (40.9%)	67 (29.3%)		0.203 ^b^
IP MI (n,%)	38 (40.9%)	119 (52%)		
NSTEMI (n,%)	15 (16.1%)	36 (15.7%)		
Other loc. (n,%)	2 (2.2%)	7 (3.1%)		
Killip I (n,%)	51 (61.3%)	160 (69.9%)		0.128 ^c^
Killip II (n,%)	28 (30.1%)	58 (25.3%)		
Killip III (n,%)	8 (8.6%)	8 (3.5%)		
Killip IV (n,%)	0	3 (1.3%)		
BBB (n,%)	7 (7.5%)	29 (12.7%)		0.185 ^b^
VF (n,%)	3 (3.2%)	12 (5.2%)		0.567 ^b^
VT (n,%)	17 (17.2%)	45 (19.7%)		0.612 ^b^
A.fib (n,%)	11 (11.8%)	17 (7.4%)		0.204 ^b^
AV block gr. I (n,%)	5 (5.4%)	19 (8.3%)		0.366 ^b^
AV block gr. II–III (n,%)	5 (5.4%)	13 (5.7%)		0.915 ^b^
**Echocardiography**				
EF (%) (mean ± SD)	47.8 + 11	49.2 ± 12.3		0.351 ^a^

MI—Myocardial infarction; DM—Diabetes Mellitus; yrs.—years; AS—Anteroseptal; IP—inferoposterior; NSTEMI—Non ST—elevation MI; loc.—localization; BBB—Bundle Brunch Block; VF—Ventricular Fibrillation; VT—Ventricular Tachycardia; A.fib.—Atrial Fibrillation; AV—Atrioventricular; EF—Ejection fraction; SD—Standard deviation; ^a^—One-Way Anova; ^b^—Pearson Chi Square; ^c^—Fisher Exact test; Superscript numbers—significant difference between examined group and group named by superscript (*p* < 0.05); Underline—significant difference between groups with Bonferroni correction (*p* < 0.016).

**Table 2:** Results of Cardiovascular Reflex Tests of the study population.

	1. MI/DM N = 93	2. MI N = 229	3. DM N = 53	*p* Value
Abnormal HGT (n,%)	69 (74.2%)	177 (77.3%)	49 (92.5%)	0.025 ^a^
OH (n,%)	18 (19.4%)	31 (13.5%)	6 (11.3%)	0.310 ^a^
DS (n,%)	75 (80.6%)	181 (79%)	50 (94.3%)	0.034 ^a^
Abnormal VM (n,%)	45 (48.4%)	111 (48.5%)	20 (37.7%)	0.351 ^a^
HRB (n,%)	69 (74.2%) ^2,3^	137 (59.8%) ^1,3^	22 (41.5%) ^1,2^	<0.001 ^a^
HRS (n,%)	70 (75.3%)	156 (68.1%)	39 (73.6%)	0.390 ^a^
Without DP (n,%)	1 (1.1%) ^2,3^	18 (7.9%)^1,3^	9 (17%) ^1,^^2^	0.002 ^a^
Early DP (n,%)	20 (21.5%)	73 (31.9%)	19 (35.8%)	0.159 ^a^
Definite DP (n,%)	71 (76.3%) ^2,3^	138 (60.3%) ^1^	25 (47.2%) ^1^	0.001 ^a^
CAN (n,%)	74 (79.6%)	166 (72.5%)	43 (81.1%)	0.239 ^a^
Score of AN (mean ± SD)	6.9 ± 1.6 ^2^	6.2 ± 1.9 ^1^	6.3 ± 2	0.011 ^b^

MI—Myocardial Infarction; DM—Diabetes Mellitus; HGT—Hand grip test; OH—Orthostatic Hypotension; DS—Sympathetic Dysfunction; VM—Valsalva Maneuver; HRB—Heart rate response to deep breathing; HRS—Heart rate response to standing; DP—Parasympathetic dysfunction; CAN—Complete Autonomic Neuropathy; AN—Autonomic Neuropathy; SD—Standard deviation; ^a^—Pearson Chi Square; ^b^—One-Way Anova; Superscript numbers—significant difference between examined group and group named by superscript (*p* < 0.05); Underline—significant difference between groups with Bonferroni correction (*p* < 0.016).

**Table 3:** Results of 24 h Holter ECG monitoring of the study population.

	1. MI/DM N = 93	2. MI N = 229	3. DM N = 53	*p* Value
Mean HR (bpm) (mean ± SD)	71.8 ± 10.9 ^3^	70.6 ± 10.4 ^3^	76.4 ± 9.4 ^1,2^	0.004 ^a^
SDNN (ms) (mean ± SD)	93.7 ± 34.6 ^1,2^	104.4 ± 39 ^1,3^	135.2 ± 35.4 ^1,2^	<0.001 ^a^
SDANN (ms) (Mdn (IQR))	79 (57–99.5) ^3^	87.5 (68–109) ^3^	124 (96–147) ^1,2^	<0.001 ^b^
RMSSD (ms) (Mdn (IQR))	41 (26.5–62)	47 (29–75.5)	47 (34.3–68)	0.226 ^b^
LF (ms^2^) Mdn (IQR)	1030 (385.3–2770.1)	1751.3 (704.5–4225.5)	1776.3 (491–3312.2)	0.034 ^b^
HF (ms^2^) Mdn (IQR)	336.1 (99.8–861.2)	441.1 (159.7–1337.5)	428.3 (169.8–1270.4)	0.118 ^b^
LF/HF (mean ± SD)	3.7 ± 1.9	3.9 ± 1.7	3.6 ± 1.6	0.415 ^a^

MI—Myocardial Infarction; DM—Diabetes Mellitus; HR—Heart rate; bpm—beats per minute; SDNN—Standard deviation of normal interval; ms—milliseconds; SDANN—Standard Deviation of the 5 min Average NN intervals; RMSSD—root mean square of successive differences; LF—Low frequency band (0.04–0.15 Hz); HF—High frequency band (>0.15 Hz); SD—Standard deviation; Mdn—median; IQR—Interquartile range (25–75%); ^a^—One-Way Anova; ^b^—Kruskal–Wallis test; Superscript numbers—significant difference between examined group and group named by superscript (*p* < 0.05); Underline—significant difference between groups with Bonferroni correction (*p* < 0.016).

**Table 4:** Results of Cardiovascular Reflex Test in the MI/DM group based on survival status at the end of follow-up.

	MI/DM Died N = 16	MI/DM Survived N = 77	*p* Value
Abnormal HGT (n,%)	11 (68.8%)	58 (75.3%)	0.549 ^a^
OH (n,%)	1 (6.3%)	17 (22.1%)	0.183 ^a^
DS (n,%)	12 (75%)	63 (81.8%)	0.503 ^a^
Abnormal VM (n,%)	12 (75%)	33 (42.9%)	0.019 ^b^
HRB (n,%)	13 (81.3%)	56 (72.7%)	0.754 ^a^
HRS (n,%)	11 (68.8%)	59 (76.9%)	0.532 ^a^
Without DP (n,%)	0	1 (1.3%)	1.000 ^a^
Early DP (n,%)	2 (12.5%)	19 (24.7%)	0.511 ^a^
Definite DP (n,%)	14 (87.5%)	57 (74%)	0.342 ^a^
CAN (n,%)	12 (75%)	62 (80.5%)	0.734 ^a^
Score of AN (mean ± SD)	6.8 ± 1.3	6.9 ± 1.6	0.893 ^c^

MI—Myocardial Infarction; DM—Diabetes Mellitus; HGT—Hand grip test; OH—Orthostatic Hypotension; DS—Sympathetic Dysfunction; VM—Valsalva Maneuver; HRB—Heart rate response to deep breathing; HRS—Heart rate response to standing; DP—Parasympathetic dysfunction; CAN—Complete Autonomic Neuropathy; AN—Autonomic Neuropathy; SD—Standard deviation; ^a^—Fisher Exact Test; ^b^—Chi Square Test; ^c^—Independent Samples T test.

**Table 5:** Selected factors associated with all-cause mortality in patients with DM following MI.

	N (%) of Patients	Mean Survival (Months) (95 CI%)	Univariable HR (95% CI)	Multivariable HR (95% CI)
**Basic characteristics of study population**				
Age (yrs.)			1.054 (0.990–1.123)	
Age (<65 yrs.)	6 (13.6%)	52.5 (47–58)	^1^	
Age (≥65 yrs.)	10 (20.4%)	49.5 (43.8–55.2)	1.630 (0.591–4.495)	
**Treatment strategy**				
Conservative treatment	14 (21.2%)	49.4 (44.4–54.3)	^1^	
Fibrinolytic therapy	2 (7.4%)	56 (50.7–61.3)	0.344 (0.078–1.516)	
**Clinical status at admission**				
AS MI	3 (7.9%)	55.6 (50.8–60.4)	^1^	^1^
IP MI	8 (21.1%)	50.7 (44.8–56.5)	2.376 (0.630–8.959)	2.351 (0.624–8.861)
NSTEMI	5 (33.3)	41.1 (28.5–53.7)	5.257 (1.251–22.088) *	6.029 (1.424–25.534) *
Other loc.	0			
Killip I	8 (14%)	52.6 (47.9–57.4)	^1^	
Killip II	4 (14.3%)	52.1 (45–59.2)	1.080 (0.325–3.592)	
Killip III	4 (50%)	37.9 (22.8–53)	3.971 (1.193–13.213) *	
**Echocardiography**				
EF (%)			0.997 (0.949–1.047)	
EF (>40%)	12 (18.2%)	50.8 (46.1–55.5)	^1^	
EF (≤40%)	4 (14.8%)	52 (44.8–59.1)	0.815 (0.263–2.526)	
**24 h Holter ECG**				
Mean Heart rate (bpm)			1.022 (0.974–1.072)	
Mean Heart rate (<60/min)	5 (33.3%)	44.8 (33.6–56.1)	^1^	
Mean Heart rate (60–80/min)	7 (11.9%)	54.1 (50–58.2)	2.872 (0.911–9.053)	
Mean Heart rate (>80/min)	4 (21.6%)	47 (35.8–58.1)	2.402 (0.700–8.249)	
**CART**				
VM (normal)	4 (8.3%)	55.5 (51.3–59.7)	^1^	
VM (abnormal)	12 (26.7%)	46.7 (40.3–53.1)	3.390 (1.092–10.521) *	3.713 (1.188–11.601) *
HRB (normal)	3 (12.5%)	53.8 (47.2–60.3)	^1^	
HRB (abnormal)	13 (18.8%)	50.3 (45.5–55)	1.456 (0.415–5.110)	
HRS (normal)	5 (21.7%)	49.3 (40.9–57.6)	^1^	
HRS (abnormal)	11 (15.7%)	52 (47.6–56.3)	0.723 (0.251–2.084)	
OH (normal)	15 (20%)	50.1 (45.6–54.6)	^1^	
OH (abnormal)	1 (5.6%)	56.4 (49.7–63)	0.323 (0.043–2.448)	
HGT (normal)	5 (20.8%)	50.9 (43.6–58.1)	^1^	
HGT (abnormal)	11 (15.9%)	51.4 (46.7–56)	0.937 (0.325–2.702)	
Without DS	4 (22.2%)	49.7 (40.8–58.7)	^1^	
With DS	12 (16%)	51.5 (47.2–55.9)	0.846 (0.273–2.629)	
Without DP	0		^1^	
With early DP	2 (9.5%)	55.3 (49.2–61.5)		
With definite DP	14 (19.7%)	49.9 (45.2–54.6)	2.279 (0.518–10.032)	

Yrs.—years; MI—Myocardial Infarction; AS—Anteroseptal; IP—Inferoposterior; NSTEMI—Non ST elevation MI; loc. localization; EF—Ejection Fraction; bpm—beats per minute; HGT—Hand grip test; OH—Orthostatic Hypotension; DS—Sympathetic Dysfunction; VM—Valsalva Maneuver; HRB—Heart rate response to deep breathing; HRS—Heart rate response to standing; DP—Parasympathetic dysfunction; CI—Confidence Interval; HR—Hazard ratio; ^1^—reference category; *—*p* values < 0.05.

## Data Availability

The original contributions presented in this study are included in the article and its Supplementary Material. Further inquiries can be directed to the corresponding author.

## Supplementary Materials

The following supporting information can be downloaded at https://www.mdpi.com/article/10.3390/medicina62010096/s1. Table S1: Basic characteristics, clinical status upon admission and EF of MI/DM group, based on survival status at the end of follow up; Table S2: Results of 24h Holter ECG monitoring in MI/DM group, based on survival status at the end of follow up are shown; Table S3: All factors associated with overall mortality in patients with DM after MI; Table S4: Factors associated with overall mortality in patients with DM after MI, with exact p-values; Table S5: Basic characteristics, clinical status upon admission and EF of MI/DM group, based on results of VM.
