# Supplementary material for: Autonomic Nervous System Dysfunction in Diabetic Patients After Myocardial Infarction: Prognostic Role of the Valsalva Maneuver
Source: Medicina (Kaunas). 2026 Jan 1;62(1):96. doi: 10.3390/medicina62010096 (PMC12843366; doi:10.3390/medicina62010096)
Supplement: Supplementary file 1 [file medicina-62-00096-s001.zip › medicina-4058588-supplementary.pdf]

Supplementary Materials

Supplementary Table S1. Basic characteristics, clinical status upon admission and EF of MI/DM group, based on survival status at the end of follow up.

	MI/DM Died N = 16	MI/DM Survived N = 77	p value
**Basic characteristics of study population**			
Age (yrs.) (mean ± SD)	66.2 ± 7	62.7 ± 8.6	.130[a]
Male (n,%)	12 (75%)	44 (57.1%)	.184[b]
Previous MI (n,%)	3 (18.8%)	10 (13%)	.691[c]
**Treatment strategy**			
Treatment strategy			
Fibrinolytic therapy	2 (12.5%)	25 (32.5%)	.138[c]
**Clinical status at admission**			
AS MI (n,%)	3 (18.8%)	35 (45.5%)	.120[c]
IP MI (n,%)	8 (50%)	30 (30.9%)	
NSTEMI (n,%)	5 (31.3%)	10 (13.3%)	
Other loc. (n,%)	0	2 (2.6%)	
Killip I (n,%)	8 (50%)	49 (63.6%)	.089[c]
Killip II (n,%)	4 (25%)	21 (31.2%)	
Killip III (n,%)	4 (25%)	4 (5.2%)	
BBB (n,%)	1 (6.3%)	6 (7.8%)	1.000[c]
VF (n,%)	0	3 (3.9%)	1.000[c]
VT (n,%)	0	16 (20.8%)	.065[c]
A.fib (n,%)	0	11 (14.3%)	.201[c]
AV block gr. I (n,%)	2 (12.5%)	3 (3.9%)	.203[c]
AV block gr. II - III (n,%)	1 (6.3%)	4 (5.2%)	1.000[c]
**Echocardiography**			
EF (%) (mean ± SD)	47.6 + 14	47.9 ± 10.3	.936[a]

MI – Myocardial infarction; DM – Diabetes Mellitus; yrs. – years; AS – Anteroseptal; IP – Inferiorposterior; NSTEMI – Non ST – elevation MI; loc. – localization; BBB – Bundle Branch Block; VF - Ventricular Fibrillation; VT – Ventricular Tachycardia; A.fib. –Atrial Fibrillation; AV – Atrioventricular; EF – Ejection fraction; SD – Standard deviation; [a]-Independent Samples T test; [b] – Pearson Chi Square; [c]- Fisher Exact test;

Supplementary Table S2. Results of 24h Holter ECG monitoring in MI/DM group, based on survival status at the end of follow up are shown

	MI/DM died N = 16	MI/DM survived N = 77	p value
Mean HR (bpm) (mean ± SD)	72.8 ± 16.4	71.6 ± 9.5	.780[a]
SDNN (ms) (mean ± SD)	88.5 ± 40	94.8 ± 33.6	.511[a]
SDANN (ms) (Mdn (IQR))	72.5 (47.8 – 93)	81 (59.5 – 102)	.297[b]
RMSSD (ms) (Mdn (IQR))	35.5 (27.5 – 47.3)	41 (26.5 - 68)	.439[b]
LF (ms^2) Mdn (IQR))	563.1 (354.7 – 1627.1)	1061.3 (440.3 – 3592.7)	.140[b]

HF (ms^2) Mdn (IQR))	176.5 (100.3 – 416)	341.8 (98.9 – 995.9)	.214[b]
LF/HF (mean ± SD)	4 (1.7 – 5.3)	3.3 (2.4 – 5.1)	.839[b]

MI – Myocardial Infarction; DM – Diabetes Mellitus; HR – Heart rate; bmp – beats per minute; SDNN – Standard deviation of normal interval; ms – milliseconds; SDANN - Standard Deviation of the 5-minute Average NN intervals; RMSSD - root mean square of successive differences; LF – Low frequency band (.04 - .15 Hz); HF – High frequency band (>.15 Hz); SD – Standard deviation; Mdn – median; IQR – Interquartile range (25%-75%); [a] – Independent Samples T test; [b]- Mann Whitney U test.

Supplementary Table S3. All factors associated with overall mortality in patients with DM after MI.

Ventricular Tachycardia (VT), Ventricular Fibrillation (VF), and A.fib. were not used for the univariable and multivariable models because none of the patients with A.fib/VT/VF had the outcome of interest.

	N (%) of patients	Univariable P value	Multivariable P value
		**Basic characteristics of study population**	
Age (yrs.)		1.054 (.990-1.123)	
Age (< 65 yrs.)	6 (13.6%)	[1]	
Age (=> 65 yrs.)	10 (20.4%)	1.630 (.591 – 4.495)	
Female	4 (10.8%)	[1]	
Male	12 (21.4%)	.540 (.174 – 1.675)	
Without Previous MI	13 (16.3%)	[1]	
With previous MI	3 (23.1%)	1.942 (.551 – 6.849)	
		**Treatment strategy**	
Conservative treatment	14 (21.2%)	[1]	
Fibrinolytic therapy	2 (7.4%)	.344 (.078 – 1.516)	
		**Clinical status at admission**	
AS MI	3 (7.9%)	[1]	[1]
IP MI	8 (21.1%)	2.376 (.630 – 8.959)	2.351 (.624-8.861)
NSTEMI	5 (33.3)	5.257 (1.251 – 22.088)*	6.029 (1.424 – 25.534)*
Other loc.	0		
Killip I	8 (14%)	[1]	
Killip II	4 (14.3%)	1.080 (.325 – 3.592)	
Killip III	4 (50%)	3.971 (1.193 – 13.213)*	
Without BBB	15 (17.4%)	[1]	
With BBB	1 (14.3%)	.871 (.115-6.611)	
Without VF	16 (17.8%)		
With VF	0		
Without VT	16 (20.8%)		
With VT	0		
Without A. Fib.	16 (19.5%)		
With A.Fib.	0		
Without AV block gr. I	14 (15.9%)	[1]	
With AV block gr. I	2 (40%)	2.351 (.534 – 10.4)	
Without AV block gr. II-III	15 (17%)	[1]	
With AV block gr. II-III	1 (20%)	.887 (.117 – 6.734)	
		**Echocardiography**	
EF (%)		.997 (.949 – 1.047)	
EF (> 40%)	12 (18.2%)	[1]	
EF (<= 40%)	4 (14.8%)	.815 (.263 – 2.526)	
		**24h Holter ECG**	

Mean Heart rate (bpm)		1.022 (.974 – 1.072)	
Mean Heart rate (<60/min)	5 (33.3%)	1	
Mean Heart rate (60-80/min)	7 (11.9%)	2.872 (.911 – 9.053)	
Mean Heart rate (>80/min)	4 (21.6%)	2.402 (.700 – 8.249)	
SDNN (ms)		.995 (.980 – 1.010)	
SDNN (=>100ms)	4 (11.1%)	1	
SDNN (<100ms)	12 (21.1%)	2.014 (.649 – 6.249)	
SDANN (ms)		1.001 (.993 – 1.009)	
SDANN (=>79 ms)	7 (14.9%)	1	
SDANN (<79 ms)	9 (19.6%)	1.368 (.509 – 3.678)	
RMSSD (ms)		.994 (.979 – 1.009)	
RMSSD (=>41 ms)	6 (12.8%)	1	
RMSSD (<41 ms)	10 (21.7%)	1.327 (.480 – 3.666)	
LF (ms^2)		1.000 (1.000-1.000)	
LF (=>1030 ms^2)	6 (13%)	1	
LF (<1030 ms^2)	10 (21%)	1.415 (.514 – 3.897)	
HF (ms^2)		1.000 (1.000-1.000)	
HF (=> 336.1 ms^2)	5 (10.6%)	1	
HF (< 336.1 ms^2)	11 (23.9%)	1.952 (.678 – 5.622)	
LF/HF		.994 (.766 – 1.272)	
LF/HF (1.5-2)	2 (50%)	1	
LF/HF (<1.5)	2 (20%)	.316 (0.440 – 2.245)	
LF/HF (>2)	12 (15.2%)	.276 (.062 – 1.236)	
		**CART**	
VM (normal)	4 (8.3%)	1	
VM (abnormal)	12 (26.7%)	3.390 (1.092 – 10.521)*	3.713 (1.188 – 11.601)*
HRB (normal)	3 (12.5%)	1	
HRB (abnormal)	13 (18.8%)	1.456 (.415 - 5.110)	
HRS (normal)	5 (21.7%)	1	
HRS (abnormal)	11 (15.7%)	.723 (.251 - 2.084)	
OH(normal)	15 (20%)	1	
OH (abnormal)	1 (5.6%)	.323 (.043 – 2.448)	
HGT (normal)	5 (20.8%)	1	
HGT (abnormal)	11 (15.9%)	.937 (.325 – 2.702)	
Without DS	4 (22.2%)	1	
With DS	12 (16%)	.846 (.273 – 2.629)	
Without DP	0	1	
With early DP	2 (9.5%)		
With definite DP	14 (19.7%)	2.279 (.518 – 10.032)	
Without CAN	4 (21.1%)	1	
With CAN	12 (16.2%)	.890 (.287 – 2.762)	
Score of AN		1.047 (.758 – 1.446)	
Score of AN (<7)	5 (14.7%)	1	
Score of AN (=>7)	11 (18.6%)	1.481 (.514 – 4.266)	

Yrs. – years; MI – Myocardial Infarction; AS – Anteroseptal; IP – Inferiorposterior; NSTEMI – Non ST elevation MI; loc. localization; BBB – Bundle Branch Block; VF – Ventricular Fibrillation; VT – Ventricular Tachycardia; A. Fib. – Atrial Fibrillation; AV – Atrioventricular; EF – Ejection Fraction; bpm – beats per minute; SDNN – Standard deviation of normal interval; ms – milliseconds; SDANN - Standard Deviation of the 5 minute Average NN intervals; RMSSD - root mean square of successive differences; LF – Low frequency band (.04 - .15 Hz); HF – Low frequency band (>.15 Hz); HGT – Hand grip test; OH – Orthostatic Hypotension; DS – Sympathetic Dysfunction;

VM – Valsalva Maneuver; HRB – Heart rate response to stading; DP – Parasympathetic dysfunction; CAN – Complete Autonomic Neuropathy; AN – Autonomic Neuropathy; [1] – reference category; * - p values < .05.

Supplementary Table S4. Factors associated with overall mortality in patients with DM after MI, with exact p-values.

	N (%) of patients	Univariable P value	Multivariable P value
**Basic characteristics of study population**			
Age (yrs)		.102	
Age (< 65 yrs)	6 (13.6%)	[1]	
Age (=> 65 yrs)	10 (20.4%)	.345	
Female	4 (10.8%)	[1]	
Male	12 (21.4%)	.286	
Without Previous MI	13 (16.3%)	[1]	
With previous MI	3 (23.1%)	.302	
**Treatment strategy**			
Conservative treatment	14 (21.2%)	[1]	
Fibrinolytic therapy	2 (7.4%)	.159	
AS MI	3 (7.9%)	[1]	[1]
IP MI	8 (21.1%)	.201	.207
NSTEMI	5 (33.3)	.023*	.015*
Other loc.	0		
Killip I	8 (14%)	[1]	
Killip II	4 (14.3%)	.900	
Killip III	4 (50%)	.025*	
Without BBB	15 (17.4%)	[1]	
With BBB	1 (14.3%)	.894	
Without VF	16 (17.8%)		
With VF	0		
Without VT	16 (20.8%)		
With VT	0		
Without A. Fib.	16 (19.5%)		
With A.Fib.	0		
Without AV block gr. I	14 (15.9%)	[1]	
With AV block gr. I	2 (40%)	.258	
Without AV block gr. II-III	15 (17%)	[1]	
With AV block gr. II-III	1 (20%)	.908	
**Echocardiography**			
EF (%)		.899	
EF (> 40%)	12 (18.2%)	[1]	
EF (<= 40%)	4 (14.8%)	.723	
**24h Holter ECG**			
Mean Heart rate (bpm)		.370	
Mean Heart rate (<60/min)	5 (33.3%)	[1]	
Mean Heart rate (60-80/min)	7 (11.9%)	.072	
Mean Heart rate (>80/min)	4 (21.6%)	.164	
SDNN (ms)		.521	
SDNN (=>100ms)	4 (11.1%)	[1]	
SDNN (<100ms)	12 (21.1%)	.225	

SDANN (ms)		.819	
SDANN (=>79 ms)	7 (14.9%)	[1]	
SDANN (<79 ms)	9 (19.6%)	.534	
RMSSD (ms)		.453	
RMSSD (=>41 ms)	6 (12.8%)	[1]	
RMSSD (<41 ms)	10 (21.7%)	.585	
LF (ms^2)		.931	
LF (=>1030 ms^2)	6 (13%)	[1]	
LF (<1030 ms^2)	10 (21%)	.502	
HF (ms^2)		.544	
HF (=> 336.1 ms^2)	5 (10.6%)	[1]	
HF (< 336.1 ms^2)	11 (23.9%)	.215	
LF/HF		.960	
LF/HF (1.5-2)	2 (50%)	[1]	
LF/HF (<1.5)	2 (20%)	.249	
LF/HF (>2)	12 (15.2%)	.092	
		**CART**	
VM (normal)	4 (8.3%)	[1]	
VM (abnormal)	12 (26.7%)	.035*	.024*
HRB (normal)	3 (12.5%)	[1]	
HRB (abnormal)	13 (18.8%)	.558	
HRS (normal)	5 (21.7%)	[1]	
HRS (abnormal)	11 (15.7%)	.548	
OH(normal)	15 (20%)	[1]	
OH (abnormal)	1 (5.6%)	.274	
HGT (normal)	5 (20.8%)	[1]	
HGT (abnormal)	11 (15.9%)	.904	
Without DS	4 (22.2%)	[1]	
With DS	12 (16%)	.773	
Without DP	0	[1]	
With early DP	2 (9.5%)		
With definite DP	14 (19.7%)	.276	
Without CAN	4 (21.1%)	[1]	
With CAN	12 (16.2%)	.840	
Score of AN		.782	
Score of AN (<7)	5 (14.7%)	[1]	
Score of AN (=>7)	11 (18.6%)	.467	

Yrs. – years; MI – Myocardial Infarction; AS – Anteroseptal; IP – Inferiorposterior; NSTEMI – Non ST elevation MI; loc. localization; BBB – Bundle Branch Block; VF – Ventricular Fibrillation; VT – Ventricular Tachycardia; A. Fib. – Atrial Fibrillation; AV – Atrioventricular; EF – Ejection Fraction; bpm – beats per minute; SDNN – Standard deviation of normal interval; ms – milliseconds; SDANN - Standard Deviation of the 5 minute Average NN intervals; RMSSD - root mean square of successive differences; LF – Low frequency band (.04 - .15 Hz); HF – Low frequency band (>.15 Hz); HGT – Hand grip test; OH – Orthostatic Hypotension; DS – Sympathetic Dysfunction; VM – Valsalva Maneuver; HRB – Heart rate response to standing; DP – Parasympathetic dysfunction; CAN – Complete Autonomic Neuropathy; AN – Autonomic Neuropathy; [1] – reference category; * - p values < .05.

.

Supplementary Table S5. Basic characteristics, clinical status upon admission and EF of MI/DM group, based on results of VM.

	MI/DM Abnormal VM N = 45	MI/DM Normal VM N = 48	P value
**Basic characteristics of study population**			
Age (yrs.) (mean ± SD)	63.8 ± 8.8	62.9 ± 8.2	.576[a]
Male (n,%)	29 (64.4%)	27 (56.3%)	.420[b]
Previous MI (n,%)	5 (11.1%)	8 (16.7%)	.440[b]
**Treatment strategy**			
Fibrinolytic therapy	16 (35.6%)	11 (22.9%)	.180[b]
**Clinical status at admission**			
AS MI (n,%)	18 (40%)	20 (41.7%)	.689[c]
IP MI (n,%)	20 (44.4%)	18 (37.5%)	
NSTEMI (n,%)	7 (15.6%)	8 (16.7%)	
Other loc. (n,%)	0	2 (4.2%)	
Killip I (n,%)	27 (60%)	30(62.5%)	.733[c]
Killip II (n,%)	13 (28.9%)	15 (31.3%)	
Killip III (n,%)	5 (11.1%)	3 (6.3%)	
BBB (n,%)	3 (6.7%)	4 (8.3%)	1.000[c]
VF (n,%)	1 (2.2%)	2 (4.2%)	1.000[c]
VT (n,%)	7 (15.6%)	9 (18.8%)	.683[b]
A.fib (n,%)	5 (11.1%)	6 (12.5%)	.836[b]
AV block gr. I (n,%)	3 (6.7%)	2 (4.2%)	.671[c]
AV block gr. II - III (n,%)	2 (4.4%)	3 (6.3%)	1.000[c]
**Echocardiography**			
EF (%) (mean ± SD)	48.8 + 11.5	46.9 ± 10.4	.420[a]

MI – Myocardial infarction; DM – Diabetes Mellitus; yrs. – years; AS – Anteroseptal; IP – Inferiorposterior; NSTEMI – Non ST – elevation MI; loc. – localization; BBB – Bundle Branch Block; VF - Ventricular Fibrillation; VT – Ventricular Tachycardia; A.fib. –Atrial Fibrillation; AV – Atrioventricular; EF – Ejection fraction; SD –Standard deviation; [a]-Independent Samples T test; [b] – Pearson Chi Square; [c]- Fisher Exact test
